# ASO Author Reflections: Pathologic Complete Response of Extended CROSS Criteria Patients with Esophageal Cancer

**DOI:** 10.1245/s10434-020-09438-x

**Published:** 2020-12-07

**Authors:** H. H. Wang, J. Th. M. Plukker, G. A. P. Hospers

**Affiliations:** 1 Department of Surgical Oncology, University of Groningen, University Medical Center Groningen, Groningen, The Netherlands; 2 Department of Medical Oncology, University of Groningen, University Medical Center Groningen, Groningen, The Netherlands

## Past

Neoadjuvant chemoradiotherapy (nCRT) followed by surgery according to the Chemoradiotherapy Regimen for esophageal cancer followed by Surgery Study (Chemoradiotherapy Regimen for esophageal cancer followed by Surgery Study, CROSS) currently is the standard treatment for locally advanced esophageal cancer (EC) (cT1/N^+^ or T2-4a/N0-3/M0). In addition to improving radical resection rates up to 92%, the CROSS regimen (carboplatin at an area under the curve [AUC] of 2 mg/mL/min and 50 mg/m^2^ of paclitaxel with concurrent radiotherapy [41.4 Gy/23 × 1.8 Gy/5 days per week] followed by surgery) has increased the 5-year overall survival (OS) rate by 13% compared with surgery alone.^1^ These results are based on the strict highly selected criteria in the randomized controlled trial (RCT) enrollment process including a potentially curable esophageal carcinoma < 8 cm long; age of 75 years or younger; adequate hematologic, renal, hepatic, and pulmonary function; WHO performance score of 2 or lower; no history of other malignancy; and < 10% weight loss.^2^ However, in the past decade, the eligibility criteria were extended in daily practice to all patients with potentially curatively resectable locally advanced ECs if fit for surgery. The large-scale effect of extending the CROSS criteria for a pathologic complete response (pCR) remains unclear. The problems addressed included the questionable consistency of defining pCR for real-world patients with extended CROSS (e-CROSS) criteria and the contradictory outcomes. The authors investigated data from the national Dutch Upper Cancer Audit (DUCA) database for EC patients treated between 2009 and 2017. The key question asked what effect extension of the CROSS criteria for both total (ypT0N0) and local (ypT0) pCR has on the surgical radicality, postoperative morbidity, and mortality of EC patients.

## Present

Overall, patients in the CROSS and e-CROSS groups had equal total and local pCR rates.^3^ However, a separate analysis assessing the difference between histologic EC subtypes showed a higher pCR for squamous cell carcinoma in the CROSS group (48.2%) than in the e-CROSS group (33.3%) (*P* < 0.000). Surgical radicality did not differ between the two groups, but the e-CROSS group had higher postoperative mortality (3.2% vs. 4.6%) and morbidity (58.3% vs. 61.8%) (*P* = 0.048). These results show that it is necessary for clinicians to consider carefully who will benefit most in the real-world setting and to focus more on personalized care for preventing complications is warranted.

## Future

The effectiveness of extending the CROSS criteria for overall survival should be further investigated. Moreover, alternative personalized treatment options should be explored for the patients with a “risk-treatment paradox,” who often are not represented in RCTs.^4^ The current analyses did not include all the CROSS criteria nor questionable resectable tumors. For an accurate determination of the impact from the extended CROSS criteria, clinicians should be more adequately informed about the real “irresectable” tumors and the effect of the individual extended CROSS criteria. Therefore, the authors recommend future external validation of their findings focused on prospective studies including more CROSS criteria and marginal resectable tumors.

## References

[1] Van Hagen P, Hulshof MCCM, Van Lanschot JJB (2012). Preoperative chemoradiotherapy for esophageal or junctional cancer. N Engl J Med..

[2] Shapiro J, van Lanschot JJB, Hulshof MCCM (2015). Neoadjuvant chemoradiotherapy plus surgery versus surgery alone for oesophageal or junctional cancer (CROSS): long-term results of a randomised controlled trial. Lancet Oncol..

[3] Wang H, de Heer EC, Hulshoff JB (2020). Effect of extending the original CROSS criteria on tumor response to neoadjuvant chemoradiotherapy in esophageal cancer patients: a national multicenter cohort analysis. Ann Surg Oncol..

[4] Kennedy-Martin T, Curtis S, Faries D (2015). A literature review on the representativeness of randomized controlled trial samples and implications for the external validity of trial results. Trials..

